# The Influence of Graft Length and Density on Dispersion, Crystallisation and Rheology of Poly(ε-caprolactone)/Silica Nanocomposites

**DOI:** 10.3390/molecules24112106

**Published:** 2019-06-03

**Authors:** Maria Eriksson, Joris Hamers, Ton Peijs, Han Goossens

**Affiliations:** 1 Laboratory of Polymer Materials, Department of Chemical Engineering and Chemistry, Eindhoven University of Technology, 5600 MB Eindhoven, The Netherlands; mailmariaeriksson@gmail.com (M.E.); jorishamers@hotmail.nl (J.H.); 2 Materials Engineering Centre (MEC), WMG, University of Warwick, Coventry CV4 7AL, UK; 3 SABIC, Plasticslaan 1, 4612 PX Bergen op Zoom, The Netherlands; Han.Goossens@sabic.com

**Keywords:** nanocomposites, polymer, silica, grafting, dispersion, rheology, crystallisation

## Abstract

Different techniques of grafting polymer chains to filler surfaces are often employed to compatibilise filler and polymer matrices. In this paper the influence of graft length and graft density on the state of dispersion, crystallisation and rheological properties of poly(ε-caprolactone) (PCL)/silica (SiO_2_) nanocomposites are reported. Grafted silica nanoparticles were prepared through polymerisation of PCL from the nanoparticle surface. Graft length was controlled by the reaction time, while the grafting density was controlled by the monomer-to-initiator ratio. Grafted nanoparticles were mixed with PCL of different molecular weights and the state of dispersion was assessed. Different matrix-to-graft molecular weight ratios resulted in different states of dispersion. Composites based on the higher molecular weight matrix exhibited small spherical agglomerates while the lower molecular weight matrix revealed more sheet-like microstructures. The state of dispersion was found to be relatively independent of graft length and density. Under quiescent conditions the grafts showed increased nucleation ability in the higher molecular weight PCL, while in the lower molecular weight matrix the effect was less pronounced. Rheological experiments showed an increase in viscosity with increased filler content, which was beneficial for the formation of oriented structures in shear-induced crystallisation.

## 1. Introduction

Due to their large surface-to-volume ratio and poor compatibility with most polymers, inorganic nanofillers are rather difficult to disperse in many polymer matrices. In these matrices, filler-filler interactions are favoured over polymer-filler interactions and filler agglomerates are readily formed. The advantage of nanofillers over more conventional micron-sized fillers is related to their small size, large surface area and small interparticle distances already at relative low filler loadings [1]. Well-dispersed nanofillers have, in several cases, shown to enhance the stiffness of polymer matrices without compromising ductility [2,3]. In addition, nanofillers can act as barriers for crack growth and thereby delay the formation of cracks large enough to initiate failure. For example, it has been shown that the toughness of polyamide 6 (PA6)/silica nanocomposites depends on the ligament thickness between nanoparticles and that above a critical ligament thickness no toughening effect was observed [4]. However, this is only true for well-dispersed nanofillers which are smaller than the critical crack size of the polymer matrix. Large fillers or agglomerates can act as stress concentrators and can, thus, deteriorate the mechanical properties [1,3]. Heavy agglomeration is, therefore, certainly not desired, since it reduces the effective surface area of the particles and increases the interparticle distance, thereby counteracting the nanosize effect. There are, however, properties—for example, electrical or thermal conductivity—where a certain degree of agglomeration, i.e., intimate contact between fillers is beneficial for optimal performance [5,6,7]. A need for controlled dispersion is, therefore, generally required in nanocomposites. In order to tune the dispersion in a given composite system, the filler-filler interactions and/or the polymer-filler interactions need to be modified.

In order to modify these interactions and, hence, the dispersion behaviour of various fillers in a polymer matrix, grafting of the same [6,8,9,10,11,12,13] or a different polymer [14,15] to the filler surface is often performed. These grafts reduce filler-filler interactions and enhance the compatibility of the filler and matrix. Hasegawa et al. [16] already showed, both experimentally and theoretically, that there is an optimum graft density for dispersing polymer grafted particles in a polymer matrix. In another interesting paper Akcora et al. [9] showed that controlled agglomeration in a nanocomposite with polystyrene (PS)-grafted silica nanoparticles in a PS matrix could be used to create different microstructures, i.e., sheets of particles, spherical aggregates, strings of particles or well-dispersed, isolated nanoparticles, depending on the ratio between the molecular weight of the matrix and the grafts and the graft density.

The influence of grafting on the properties of the obtained nanocomposites is often studied for amorphous polymers. Various groups studied the influence of grafting on the glass transition temperature [13], mechanical properties [10] and rheological properties of nanocomposites [11,12,17]. Oh and Green showed that the glass transition temperature (*T_g_*) could be increased (long grafts) or decreased (short grafts) depending on the graft length of PS grafts in the nanocomposite, in which PS-grafted gold (Au) nanoparticles were dispersed in a PS matrix [13]. A key question to answer here is whether the length of the grafts exceed the critical entanglement molecular weight of the bulk matrix. Akcora et al. showed that for poly(methyl methacrylate) (PMMA) nanocomposites the rheological behaviour depends on the difference in molecular weight between grafts and bulk matrix [11]. Nanocomposites based on a matrix with a higher molecular weight than the grafts, showed agglomerated particles and solid-like rheological behaviour at high filler loadings. However, in matrices with a lower molecular weight than the grafts, fillers were well dispersed and no sign of a solid-like behaviour was observed, not even at the highest filler loadings. 

For semi-crystalline polymers, there are fewer examples of the influence of grafting on nanocomposite properties, and even less on the importance of graft length and density. In the current study poly(ε-caprolactone) (PCL) was used as the matrix material. The chemistry of grafting PCL to silica particles [18,19,20], is well studied and it was shown that the addition of grafted silica particles can enhance nucleation [21] and improve mechanical properties [22] of PCL/silica nanocomposites. This nucleation effect was also observed in other PCL/nanofiller systems. For instance, Zhou et al. [23] showed that grafted carbon nanotubes have a stronger nucleating effect than their non-grafted counterparts. The effect was larger in samples with a higher grafting density and shorter grafts as compared to samples with a low grafting density and longer grafts. L’Abee et al. [24] showed that, during the in situ preparation of rubber particle-filled PCL, the peroxide used to cross-link the rubber network also caused grafting of PCL chains onto the rubber particles. Transmission electron microscopy (TEM) images of the grafted particles revealed lamellae growing from the surface of the particles, while also an increase in nucleation efficiency was observed which was shown to depend on grafting density [24]. The grafted chains also influenced the mechanical behaviour of these PCL/rubber blends. 

There are generally two strategies in grafting chemistry: the “grafting-to” [22] and the “grafting-from” approach [18,19,20]. In the “grafting-to” approach, chains of the polymer are attached to the particle surface via a coupling reaction, while in the “grafting-from” approach the polymerisation is performed in the presence of particles and an increase of the graft length with reaction time is observed [2]. Both procedures have their obvious advantages. In the “grafting-to” approach the molecular weight and the polydispersity of the chains can be determined prior to the coupling reaction, but there are limitations to the number of chains that can be added to the particles, especially if the size of the particles is small, due to the bulky size of the polymer chains. The “grafting-from” approach offers more possibilities of controlling the grafting density by tuning the number of initiating sites on the surface of the particles. Here, the limitation is that it has been difficult to determine the exact graft length and density on the particles, which complicates the analysis of the effect of the grafting on the properties of the obtained composite materials, especially if several graft lengths are to be used.

In the current study we focus on the “grafting-from” approach of PCL from a silica (SiO_2_) nanoparticle surface. This reaction is well described in the literature, both directly from the silica surface [18,19] and by using silane spacers [19,20]. First, nanoparticles with different graft lengths and densities where prepared and subsequently these particles were mixed with the PCL matrix. Properties of the resulting nanocomposites will be discussed in terms of the influence of graft length and graft density on the state of dispersion, crystallisation and rheological behaviour.

## 2. Experimental

### 2.1. Materials

Colloidal silica suspension (ORGANOSILICASOL™) in isopropanol (IPA-ST) and toluene (TOL-ST) containing 40 wt% SiO_2_ nanoparticles (10–15 nm) was obtained from Nissan Chemical Industries (City, Japan). The silica particles were prepared in ethanol using the well-known Stöber method, surface modified and transferred to the mentioned solvent. ε-Caprolactone from Sigma-Sigma-Aldrich Chemie B.V. (Zwijndrecht, Netherlands) was dried on molecular sieves prior to use. Toluene, hexane and ethanol were supplied from Biosolve B.V. (Valkenswaard, Netherlands). The toluene was distilled and stored on molecular sieves before use. Furthermore, ninhydrin (ACS reagent) and stannous octoate from Sigma-Aldrich Chemie B.V., and 3-aminopropyltriethoxysilane (97%) from abcr GmbH (Karlsruhe, Germany), were used. Two grades of poly(ε-caprolactone) were used as matrices. CAPA^®^ 6400 (M_n_ = 40 kg/mol) and CAPA^®^ 6800 (M_n_ = 80 kg/mol) were kindly provided by Perstorp Polycaprolactones (Perstorp, Sweden). These polymers are further referred to as PCL40 and PCL80, respectively. Both polymers were used as received.

### 2.2. Sample Preparation

**Grafting experiments:** First, the silica nanoparticles were silanised with a 3-aminopropyltriethoxysilane (APTES). For the reaction 4 wt% silica in toluene solution was used in a 2:1 ratio of silane-to-hydroxyl groups. The reaction was performed at 70 °C for 20–24 h and the reaction mixture was thereafter centrifuged at 10,000 rpm for 25 min, re-dispersed and centrifuged two more times before drying for 5 h at 80 °C in vacuum in order to remove residual solvent. A ninhydrin test was performed in order to determine the presence of amine groups on the silica particles after the centrifugation and drying step. A 0.4 wt% solution of ninhydrin in water was used. One millilitre of this solution was added to approximately 2 mg of sample and the solution was heated to the boiling point for about 20 s. The presence of amines could then be verified by the appearance of a blue colour. From the difference in weight loss during thermogravimetric analysis (TGA) experiments between the reacted and unreacted particles the approximate number of amino groups per square nanometre of particle surface was calculated. 

**Figure 1:**
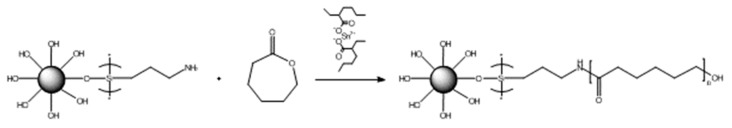
A scheme of the PCL grafting reaction.

These APTES-grafted particles were subsequently used in PCL grafting reactions with a Sn(Oct)_2_ catalyst. The reactions were performed with initiator-to-catalyst molar ratios between 1:0.33 and 1:1 in a 1 wt% solution of particles in dried toluene. The temperature was set to 100 °C and the reaction time was varied in order to vary the grafting length. Varying the initiator-to-catalyst molar ratio the grafting density could be changed. A scheme of the performed reaction is presented in Figure 1.

Details of the different grafted nanoparticles are presented in Table 1. After the grafting reaction, the particles were separated from the reaction mixture by three successive centrifugation (3000 rpm, 30 min) and re-dispersion cycles in hexane. The complete removal of unreacted monomer was confirmed by ^1^H NMR spectroscopy.

**Nanocomposite preparation:** The composites were prepared by using two different PCL grades of different molecular weight (PCL40 and PCL80) and silica nanoparticles with three different graft lengths (short, medium, long) and two different target densities (normal and half). Reference PCL samples and samples with unmodified silica particles were also prepared. The composites prepared with the PCL40 matrix were made by first dispersing the required amount of silica nanoparticles to obtain a final concentration of 1, 3 and 5 wt% of silica and 7 g of polymer in toluene. This solution was then solvent casted, left to evaporate in a fume hood overnight and subsequently dried in a vacuum oven at 40 °C overnight. Special care was taken to ensure that no residual solvent was present in the nanocomposite films after drying [25]. The dried samples were then melt-compounded in a recirculating Xplore 15MC (Sittard, Netherlands) micro-compounder at 100 °C, using a mixing time of 10–15 min and a screw speed of 50–60 rpm. For samples based on the PCL80 matrix only 1 wt% of filler was used, but the procedure was the same. 

### 2.3. Characterisation Methods

^1^H Nuclear magnetic resonance (NMR) spectroscopy was performed on a Varian Mercury 400 MHz NMR (Varian Inc., Palo Alto, CA, USA) in CDCl3 to assess if separation of the particles from the reaction mixture after centrifugation was successful. 

Fourier transform infrared spectroscopy (FTIR) analysis was performed on a Varian 3100 FTIR spectrometer (Varian Inc., Palo Alto, CA, USA) equipped with a golden gate diamond ATR setup using a spectral range between 600 and 4000 cm^−1^ and 50 scans per spectrum were co-added. 

Thermogravimetric analysis (TGA) was performed on a Q500 from TA Instruments Inc. (New Castle, DE, USA) to quantify the amount of grafted material on the modified silica nanoparticles. For this the samples were heated at 10 °C/min from 30–100 °C, kept isothermally at 100 °C for 30 min and then further heated to 800 °C. All the measurements were performed using nitrogen gas (N_2_) as a purge flow. 

Scanning electron microscopy (SEM) imaging was performed on samples which were dried and cut at −100 °C using a glass-knife and a Leica Ultracut S/FCS microtome (Leica Mikrosysteme GmbH, Wetzlar, Germany). Samples were glued onto a SEM stub and coated with a thin gold layer using an Emitech K575X sputter coater (Emitech SA, Montigny-le-Bretonneux, France). Samples were thereafter examined in a SEM model XL30 FEG from FEI Company (Eindhoven, Netherlands). 

Size exclusion chromatography (SEC) was performed on solutions of 1 mg/mL polymer using tetrahydrofuran (THF) as a solvent. Molecular weight distributions both before and after drying were determined using a SEC from Waters (Elstree, UK) with a Waters 510 pump and a Waters 712 WISP chromatograph with an injection volume of 50 μL. The column used was a PL-gel mix D column from Polymer Laboratories Ltd (Church Stretton, UK). Molecular weights were calculated relative to PS standards and are, thus, not absolute values for PCL. 

Polarized optical microscopy (POM) was used to study isothermal crystallisation behaviour and involved the use of a Zeiss Axioplan 2 optical microscope (Carl Zeiss AG, Oberkochen, Germany) equipped with a Zeiss Axiocam camera and polarisers. Thin films of nanocomposite samples placed between two glass slides were prepared with a Tribotrek. During imaging the samples were fixed in a Linkam THMS-600 hot-stage for temperature control (Linkam Scientific Instruments Ltd, Epsom, UK). 

Transmission electron microscopy (TEM) was used to study the state of dispersion of the silica particles in the polymer matrix. Ultrathin sections (70 nm) were microtomed at −100 °C using a Leica Ultracut S/FCS microtome. These sections were placed on a 200 mesh copper grid with a carbon support layer. The sections were subsequently examined in a FEI Tecnai 20 transmission electron microscope (FEI Company, Eindhoven, Netherlands), operated at 200 kV. 

Differential scanning calorimetry (DSC) using a Q1000 DSC from TA Instruments Inc. (New Castle, USA) was used to study the thermal behaviour of the PCL/silica nanocomposites. Standard aluminium pans were used and a typical sample weight was 3–5 mg. The samples were first heated to 100 °C and equilibrated for 5 min in order to remove thermal history. Subsequently the samples were cooled at 10 °C/min to −80 °C where they were kept for 10 min. Subsequently, they were reheated to 100 °C at 10 °C/min and kept isothermally for 5 min and then cooled again to −20 °C at 10 °C/min. For the isothermal crystallisation experiments, the samples were cooled at 10 °C/min to the desired temperature and kept isothermally for at least 30 min (or 90 min at the highest temperatures). Avrami analyses were performed for all measurements and PCL crystallinity was determined using a heat of fusion at 100% crystallinity of 136 J/g [26]. 

Rheometry was performed using a stress-controlled AR-G2 rheometer from TA Instruments Inc. (New Castle, USA) under nitrogen atmosphere. Different methods were used for the PCL40 and PCL80 samples. The PCL40 based samples were measured using a 25 mm plate-plate geometry and frequency sweeps were performed at 60 and 100 °C in an angular frequency range of 0.1–100 rad/s with a constant strain of 10%. Isothermal crystallisation experiments were conducted using the rheometer for the PCL40 composites. This was done using a Peltier plate assembly with a 20 mm top plate. The samples were first heated to 80 °C for 5 min and then cooled at 30 °C/min to 47 °C. Two measurements were performed for each composite, one time sweep directly upon cooling and one time sweep after applying a shear of 30 s^−1^ to the cooled sample for three seconds. Time sweeps were performed with a deformation of 1% and an angular frequency of 1 rad/s.

## 3. Results and Discussion

In the first part of this section the outcome from the silanisation and grafting reactions is presented followed by a discussion on the properties of the prepared nanocomposites and the effect of graft length.

### 3.1. Grafting Reactions

**Figure 2:**
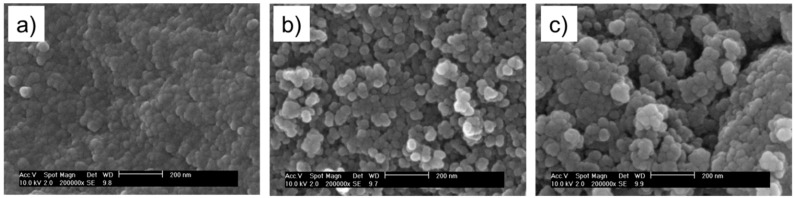
SEM images of: (**a**) silica, (**b**) silanised silica, and (**c**) grafted silica nanoparticles.

In Figure 2 SEM images of dried silica, silanised silica and grafted silica nanoparticles are shown. From these images it can be seen that the silanisation was successful. The dried silica particles tend to agglomerate very strongly into solid-like structures, but with the silane attached to the surface, this strong agglomeration is prevented and separate nanoparticles are observed. After grafting, the particles show a similar particle-like morphology, although a tendency to agglomerate into larger clusters than in the case of silanised nanoparticles can be observed.

**Figure 3:**
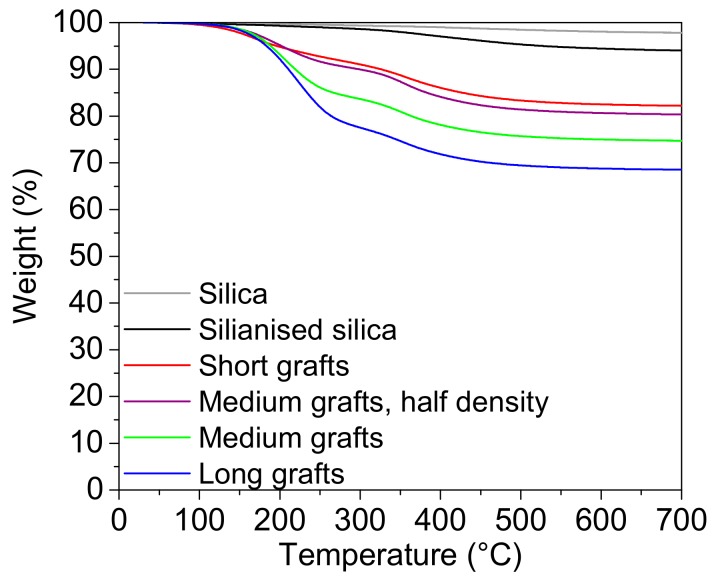
Result from the TGA experiments. Weight is plotted as a function of temperature for untreated silica, silanised silica and different types of grafted silica nanoparticles (short, medium, and long grafts).

The amount of silane and grafted polymer on the particles was estimated from TGA data. The difference in weight loss between unreacted and silanised silica particles was always around 3–4 wt%, which corresponds to a density of about 0.5–0.8 silanes per square nanometre. The TGA results for the grafted particles are shown in Figure 3. From this data the relative weight of the grafts attached to the filler surface was estimated, see Table 2. Since the amount of polymer on the surface increased with reaction time when the other reaction parameters were kept constant, it was assumed that the graft length increased with increasing reaction time. In a similar way, a reduction of the initiator-to-catalyst ratio, while keeping the other parameters constant, results in fewer polymer chain ends attached to the surface. Hence, the grafting density was reduced. Unfortunately, the length of the grafts could not be determined in a quantitative way, but the TGA experiments showed that an increased reaction time lead to increased weight of grafted material for a constant number of initiating sites, an indirect proof that graft length was indeed increased. We will further refer to them simply as short, medium and long grafts. 

**Figure 4:**
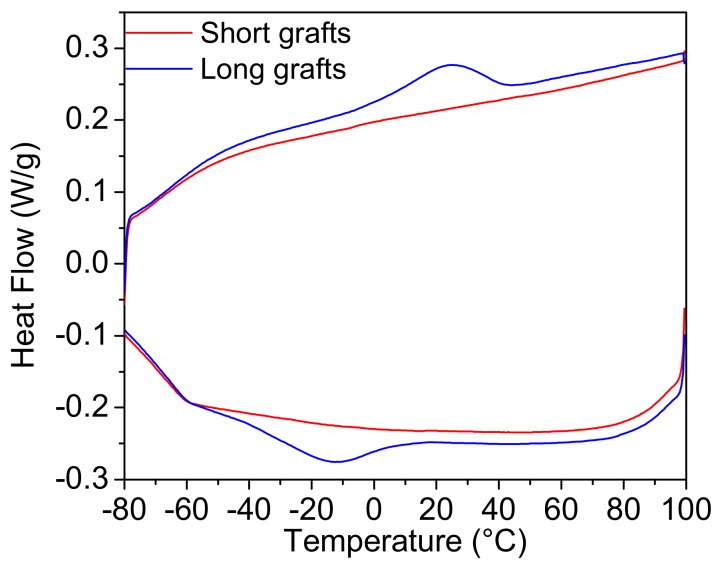
DSC traces of the cooling and the 2nd heating run for silica nanoparticles with short and long graft lengths.

The DSC traces for the cooling runs from the melt and the 2nd heating runs of two different grafted particles are presented in Figure 4. Upon cooling, a crystallisation peak is observed for the long grafted particles and upon heating the corresponding melting occurs. Particles with short grafts showed neither a melting nor a crystallisation peak. In order to fold into crystalline lamellae, the polymer chains need to have a certain minimal chain length. From the DSC experiments it is evident that the short grafts are not long enough to crystallise, while the long grafts are sufficiently long enough to crystallize. It has to be noted, however, that both the crystallisation and melting temperatures are lower than the crystallisation and melting temperatures of the neat PCL matrix, which is normally situated around 35–55 °C. This indicates that crystallisation is not favourable and that the formed crystals are not very stable. However, considering these results, it can be expected that the longer grafts will be favourable for nucleation during crystallisation of the nanocomposites.

### 3.2. Dispersion and Morphology

**Figure 5:**
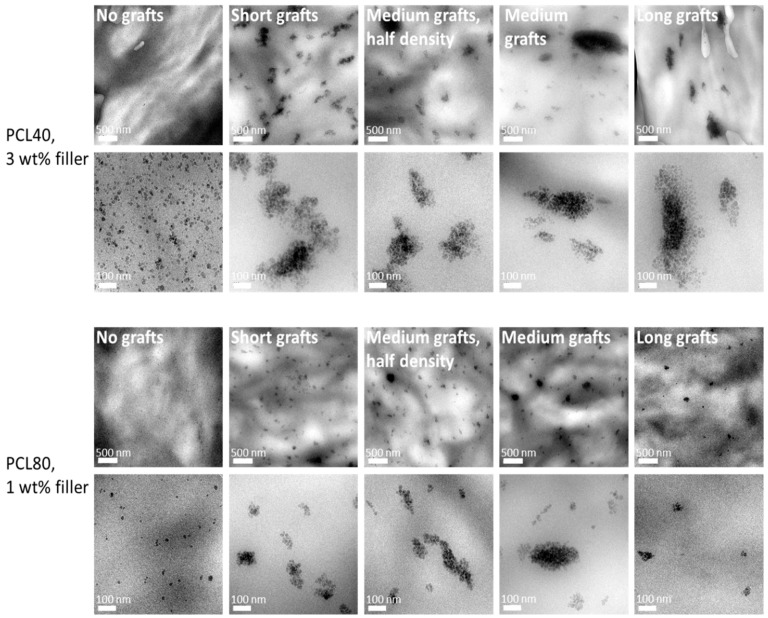
TEM images of PCL/silica composites based on PCL40 matrix and 3 wt% filler (top two rows), and PCL80 matrix and 1 wt% filler (bottom two rows) at two different magnifications. For each top row the scale bar is 500 nm and for each bottom row it is 100 nm.

The grafted silica particles were re-dispersed in toluene and mixed with PCL’s of two different molecular weights (40 and 80 kg/mol). In addition the samples were also extruded in the micro-compounder to improve the homogeneity of the dispersion. TEM pictures of the obtained morphologies are shown in Figure 5. Morphologies of 3 wt% silica in PCL40 (top row) and 1 wt% silica in PCL80 matrix (bottom row) are depicted. It can immediately be observed that the dispersion of the unmodified silica is very similar for both polymer matrices. Most of the silica nanoparticles are well dispersed in the matrices and very few agglomerates, if any, can be seen. However, dispersion is very different for the grafted silica particles. In both matrices agglomerates are found, although they are generally smaller in the PCL80 system as compared to the PCL40 matrix. There can be various explanations for this observation. One could be attributed to the higher viscosity of the PCL80 system and the development of higher shear forces during the extrusion compounding, leading to better dispersions, in other words, a kinetically driven process. The observed dispersion can also be discussed in terms of relative graft lengths, i.e., a thermodynamically governed process. Akcora et al. [9] showed, both by simulation and experiments, that PS-grafted silica particles in a PS matrix self-ensemble into different structures depending on the relative graft length and graft density. Keeping the grafting density constant while increasing the grafted chain length the particles would first form spherical aggregates followed by more sheet-like and thereafter string-like structures. Only when the graft length was high enough, the particles would be well dispersed throughout the matrix. In a more recent paper on a similar system, Chevigny et al. [12] showed that at a fixed grafting density a crossover ratio exists, where nanoparticle dispersion changes from an agglomerated to a dispersed state. In their case this crossover point could be found at a graft-to-matrix chain length ratio of around 1:4. One obvious difference between our PCL based systems and their systems is the hydrophobic nature of the PS matrix. Due to the hydroxyl groups present on the silica surface, the silica nanoparticles have a strong tendency to agglomerate in hydrophobic matrices. Therefore, well-dispersed nanoparticles could only be obtained in the PS matrix if the molecular weight of the grafted chains is much higher than the molecular weight of the matrix, i.e., a so-called wet brush regime is required. In the PCL matrix, the non-grafted particles are better dispersed than all the grafted particles due to the more hydrophilic nature of PCL. Moreover, considering the DSC results, it is reasonable to assume that the grafted chains in our work are of much lower molar mass than both PCL matrices. Therefore, a certain level of agglomeration can be expected. Increasing the graft lengths did not lead to the formation of different microstructures, since all the graft lengths are relatively short compared to the matrix molar mass. Increasing the molecular weight of the matrix, however, does have the same effect as decreasing the relative grafting length. Therefore, two different morphologies are observed. In the PCL80 matrix small spherical agglomerates can be found, while in the PCL40 matrix the agglomerates are larger and more sheet-like. It must also be mentioned that in all grafted samples some very large agglomerates can be observed. These are formed during the drying step of the grafted nanoparticles, which was necessary in order to analyse them. In the subsequent extrusion compounding step, the shear forces were not high enough to break up all these large agglomerates and, therefore, some of them where still present in the prepared composites. A more ideal way of preparing the nanocomposites might be to add the matrix polymer to the grafted particles in solution directly after polymerization and subsequently evaporate the solvent. The disadvantage of this method is that less information on the grafting reaction can be obtained and, therefore, it was chosen to dry the grafted silica particles prior to composite preparation. The similar state of dispersion of different composites made with the same PCL matrix allows us to study the effect of graft length on crystallisation behaviour in a more straightforward way. Thus, changes in crystallisation behaviour can be directly related to the graft length and not to morphological changes induced by differences in graft lengths.

### 3.3. Crystallisation Behaviour

#### 3.3.1. Non-Isothermal Crystallization

**Figure 6:**
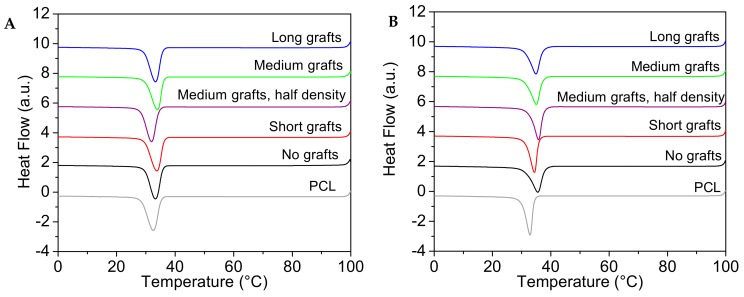
DSC cooling traces for PCL/silica composites based on PCL40 matrix and 1 wt% filler (**A**) PCL/silica composites based on PCL80 matrix and 1 wt% filler (**B**).

The crystallisation behaviour of the different PCL/silica composites was investigated using DSC and polarised optical microscopy (POM). In Figure 6 typical cooling traces from DSC experiments are presented. All PCL40 samples show a single crystallisation peak with a maximum around 32 °C. From the value of the onset of crystallization, the nucleation efficiency of the filler can be calculated according to the method first proposed by Fillon et al. for the crystallisation of polypropylene (PP) [27,28]. The maximum temperature for nucleation was determined from self-nucleating experiments and was found to be 46.3 and 45.0 °C, respectively. The crystallisation parameters extracted from the DSC traces can be found together with the nucleation efficiency (N.E.) in Table 3.

The crystallization behaviour of the PCL40 matrix is rather unaffected by the addition of small amounts of untreated nanoparticles. However, the addition of 5 wt% unmodified silica particles to the PCL40 matrix gives a negative nucleation efficiency. This is in line with our earlier data [29] where it was shown that the addition of silica nanoparticles to the same PCL40 matrix hindered crystallisation in continuous cooling experiments. Adding particles with short grafts yields a small nucleation effect, while the addition of nanoparticles with longer grafts is again disadvantageous. Reducing the grafting density is reducing the nucleation efficiency further. This result is in line with the result of L’Abee et al. [24], who showed that in a rubber particle-filled PCL based system, the nucleation efficiency depended on grafting density. For the PCL80 matrix the behaviour is different. The addition of even a small amount of 1 wt% silica nanofiller has a significant nucleating effect on the PCL matrix, evidenced by a shift in the crystallization peaks to higher temperatures. There is a clear effect of grafting length, where shorter grafts in this case are giving lower nucleation efficiency. Interestingly, this is also in line with previous data from our group for another composite system, where cellulose nanocrystals were grafted with either short or long grafts [30]. It was shown that short grafts had a better nucleation ability in the lower molecular weight matrix while longer grafts were rendering a higher nucleation efficiency in the higher molecular weight matrix. The highest nucleation efficiency is found in the sample with non-grafted particles. This can likely be attributed to the better dispersion in this sample. Moreover, a clear influence of graft length can be seen, i.e., the longer the grafts, the better the nucleation efficiency. The data also shows that a decrease in graft density is beneficial for the nucleation efficiency. The melting temperature and degree of crystallinity are rather unaffected by filler addition and the obtained crystallinity is lower for composites based on the PCL80 matrix.

#### 3.3.2. Isothermal Crystallisation 

The kinetics of isothermal crystallisation was studied using DSC and POM. It should be noted that the temperature window where the crystallisation experiments can be performed is rather limited. At too high temperatures, i.e., above 47 °C, the crystallisation takes a very long time and experiments are extremely time consuming. At too low a temperature the crystallisation starts during the cooling to the desired temperature and the determination of crystallisation parameters becomes inaccurate. With the addition of a nucleating agent the lower temperature limit is pushed upwards and the range in which the crystallisation kinetics can be compared becomes very narrow. Therefore, the crystallisation experiments were performed at different temperatures for the different composites and data sets in which the crystallisation started before the isothermal crystallization temperature was reached were not used. Avrami analysis was performed on all data sets obtained from the DSC measurements. In its simplest form the crystallisation can be described by

(1) $$X\left(t\right)=\;1-e^{⎣-K\left(T\right)\cdot t^{n}⎦}$$

where the Avrami parameters *n* and *K(T)* can be determined from the slope and the intercept of the linear fitting of log(-ln(1-X(t)) versus log(t), respectively. In accordance with literature, only values between 0.3 and 20% of relative crystallinity were used for the determination of this parameter [31] After the values of *n* and *K(T)* are obtained, the half-time of crystallisation can be calculated via:

(2) $$\tau_{0.5}={\left(\frac{ln2}{n\cdot K\left(T\right)}\right)}^{\frac{1}{n}}.$$

**Figure 7:**
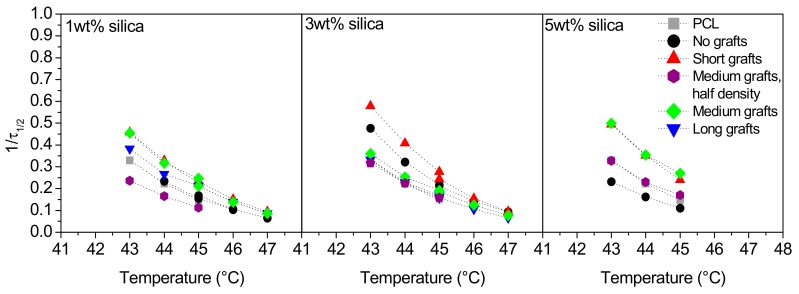
The inverse of half-time of crystallisation versus crystallisation temperature for PCL/silica composites based on PCL40 matrix and 1, 3 and 5 wt% filler (left to right).

The rate of crystallisation can be described as the inverse of the half-time of crystallisation. The rate of crystallisation at different temperatures in nanocomposites based on the PCL40 matrix can be found in Figure 7. At higher crystallisation temperatures, the crystallisation rate is very similar for all samples and very slow. At the lowest temperature, we can see that the addition of non-grafted silica particles initially accelerates the kinetics, but at high filler loading the crystallisation is, instead, slower than for the other samples. These results are coherent with the nucleation results, showing that too much well-dispersed filler particles hinders the crystal growth process. Generally, the crystallization rate is increased with increased amount of silica particles. In accordance with the non-isothermal studies, short and medium grafts have the largest positive effect on crystallisation kinetics.

**Figure 8:**
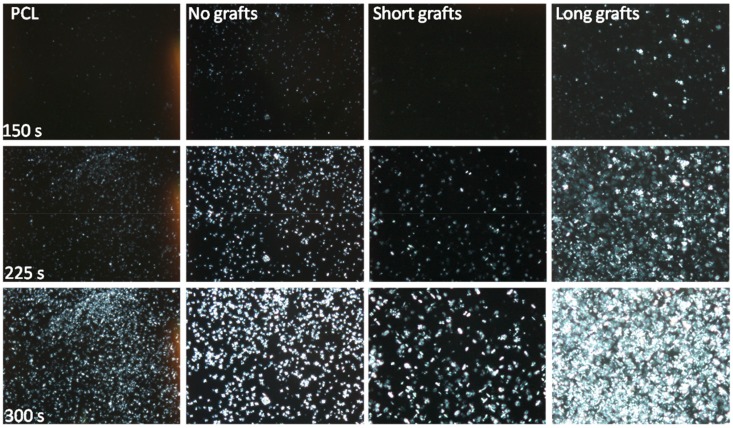
POM images of isothermal crystallisation at 45 °C for different PCL/silica composites based on the PCL40 matrix and 1 wt% filler.

In addition to the DSC experiments, isothermal crystallisation experiments were also performed under POM using a Linkam hotstage. These crystallisations were performed at 45 °C for composites based on the PCL40 matrix and the results are depicted in Figure 8. In these images the nucleation ability of the non-grafted silica nanoparticles is clearly visible. Moreover, the dependence of crystallisation kinetics on graft length is clearly shown. Particles with long grafts show a much faster initial crystallisation than their shorter grafted counterparts. At 45 °C, the isothermal DSC results indicated that there is no large difference in crystallization rate between the different grafted particles, thus, these results are somewhat contradictory to the DSC results. 

**Figure 9:**
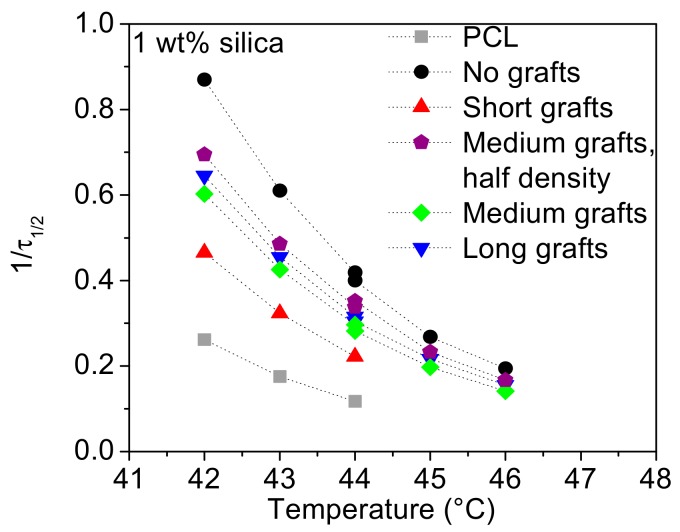
The inverse of half-time of crystallisation versus crystallisation temperature for PCL/silica composites based on the PCL80 matrix.

For the PCL80 matrix the results from the isothermal crystallisation experiments are different, but still coherent with the results of the non-isothermal crystallization. The rate of crystallisation versus crystallisation temperature can be found in Figure 9. For all filler types the crystallisation kinetics is accelerated over the whole temperature range with the effect being the largest for non-grafted silica. Shorter grafts are less efficient in accelerating the crystallisation than other fillers, while there is no large difference between medium or long grafts. Moreover, a change in graft density does not have a significant influence.

**Figure 10:**
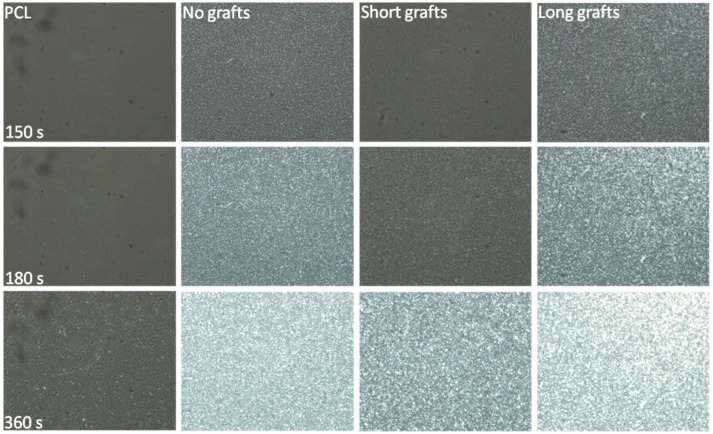
POM images taken during isothermal crystallisation at 44 °C for different PCL/silica composites based on the PCL80 matrix and 1 wt% filler.

POM images of samples based on a PCL80 matrix crystallised at 44 °C can be found in Figure 10. These results are in line with the DSC results and confirm the nanofiller’s nucleation ability. The influence of graft length is also clearly visible. Longer grafts accelerate the crystallisation to a much larger extent. Interesting to note is that these results are coherent with the results for the PCL40 matrix, even though the DSC results for those samples indicate a slightly different dependence.

From the crystallisation experiments, it can be concluded that the state of dispersion is an important factor governing the crystallisation behaviour of the nanocomposites. Generally, well-dispersed fillers show the highest nucleation efficiency and accelerate the crystallisation to the largest extent. The addition of too much well dispersed nanofiller is, however, not beneficial for both the crystallisation and nucleation efficiency and the overall rate of crystallisation decreases for filler contents exceeding 3 wt%. The grafting of PCL chains to the silica nanoparticles induces agglomeration but to a different extent in the PCL40 and PCL80 matrices. In the PCL40 matrix the large agglomerates yield a very limited nucleation efficiency during continuous cooling experiments, while they seem to accelerate crystallisation during isothermal crystallisation experiments. The influence of graft length is not clearly understood, since some of the employed experimental techniques show different trends. For the PCL80 matrix, the trend is, however, more coherent. Here, grafting of the particles leads to the formation of small silica clusters in the polymer matrix. Even though the nucleation efficiency and the rate of crystallisation is lower for the grafted particles as compared to the non-grafted samples a clear trend can be found in the influence of graft length. Increasing the graft length increases both the nucleation efficiency and the rate of the crystallisation. Decreasing the grafting density does not influence the rate of crystallisation, while the nucleation efficiency decreases. 

### 3.4. Rheological Behaviour

#### 3.4.1. Storage, Loss Modulus and Viscosity

**Figure 11:**
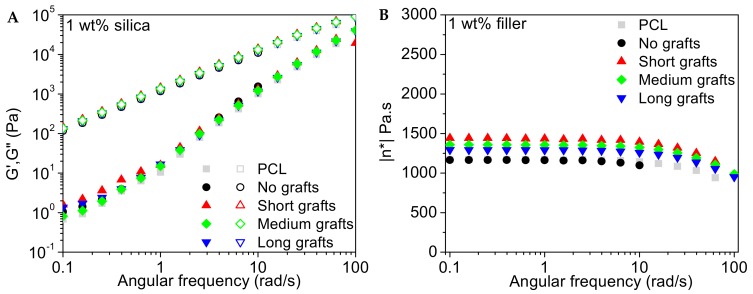
Storage and loss modulus (**A**) and complex viscosity (**B**) as a function of angular frequency for different PCL/silica composites based on the PCL40 matrix and 1 wt% filler. Measurements were performed at 60 °C.

**Figure 12:**
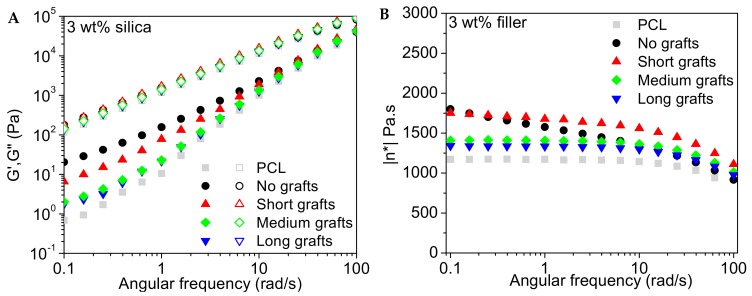
Storage and loss modulus (**A**) and complex viscosity (**B**) as a function of the angular frequency for different PCL/silica composites based on the PCL40 matrix and 3 wt% filler. Measurements were performed at 60 °C.

**Figure 13:**
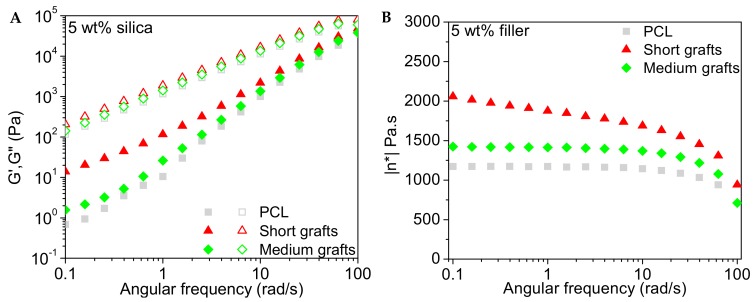
Storage and loss modulus (**A**) and complex viscosity (**B**) as a function of angular frequency for different PCL/silica composites based on the PCL40 matrix and 5 wt% filler. Measurements were performed at 60 °C.

The frequency dependence of the storage and loss modulus of the PCL40 based composites are presented in Figure 11, Figure 12 and Figure 13. The influence of the addition of 1 wt% of silica filler is very marginal for all investigated nanocomposites. At higher filler loading an increase in the storage modulus in the low frequency regime is observed, indicating that the silica nanofillers form a network within the polymer matrix. The loss modulus is less sensitive to filler addition and only at the highest filler content a modest increase is observed.

The complex viscosity as a function of angular frequency for the different composites is also presented in the same graphs. It is observed that the relatively low molecular weight of the matrix implies that the viscosity of this material is rather low and quite insensitive to shear thinning in the investigated frequency range. Only at the highest frequencies shear thinning is observed. The addition of 1 wt% silica leads to a minor increase in viscosity but the melt still follows a Newtonian-like behaviour with a close to frequency independent viscosity in the low frequency regime. At higher filler loadings a build-up of the viscosity and a change in slope of the viscosity curve at lower frequencies is observed. This change in slope points to network formation in the melt, which can be attributed to particle-particle interactions. Samples with short graft lengths show the highest increase in viscosity. This might be explained by the difference in molecular weight between the grafts and the bulk matrix. Particles with longer grafts, but still short chain lengths compared to the matrix, contribute to the large amount of low molecular weight PCL in the system, counteracting the effect of the rigid silica fillers. Hence, the effect of filler addition is less pronounced. The data for the PCL80 composites (not shown here) indicates that the addition of 1 wt% filler to the matrix does not alter the properties of the melt significantly, independent of graft length. The viscosity also remained relatively unaffected with only a minor increase in the low frequency regime for the longest grafts. 

#### 3.4.2. Shear-Induced Crystallisation 

Processing of polymers typically involves flow of a polymer melt followed by a solidification step. We have shown that the addition of various grafted silica nanoparticles affects both the melt properties and the solidification step, in this case crystallisation. Strong flow fields and long relaxation times can promote the crystallisation of oriented structures in the polymer. It is well known that shear can induce crystallisation via the alignment of chains in the direction of shear flow. These aligned chains will form nuclei for the crystallisation and thereby accelerate the crystallization process. If crystallisation takes place under conditions where chain relaxation is slow, row-like nuclei are formed, which promotes the crystallisation of oriented structures, i.e., so-called shish-kebab structures [32]. If the chains are relaxed before the crystallisation sets in, point-like nuclei will be formed. Both types of nuclei accelerate the crystallisation process. The understanding of the influence of fillers on shear-induced crystallisation is not straightforward since several different phenomena are involved. In most cases the addition of rigid fillers leads to an increase in viscosity and decrease in mobility in the polymer melt. This might have advantageous or disadvantageous effects on shear-induced crystallization. On one hand, reduced chain mobility can obstruct the orientation of polymer chains in the direction of shear flow. If the applied shear is not severe enough, fillers remain randomly distributed in the polymer melt and counteract the alignment of the polymer chains, thereby impeding the formation of shear-induced nuclei [30]. However, if the shear flow is sufficiently strong to orient fillers in the direction of flow, they can enhance the nucleation effect through slowing down the relaxation process of the polymer chains after the shear has been removed, with more polymer chains becoming nuclei [33]. In a previous paper we showed that grafted particles affected the polymer melt in a different way depending on graft length [29]. Cellulose nanocrystals with short grafts promoted nucleation with and without shear. However, the grafts hindered the orientation of the filler in the direction of flow, and the resulting crystals where non-oriented spherulites. However, long grafts, but still short in comparison to the bulk matrix, reduced the overall viscosity of the melt and, therefore, also reduced the effect of shear [30]. 

**Figure 14:**
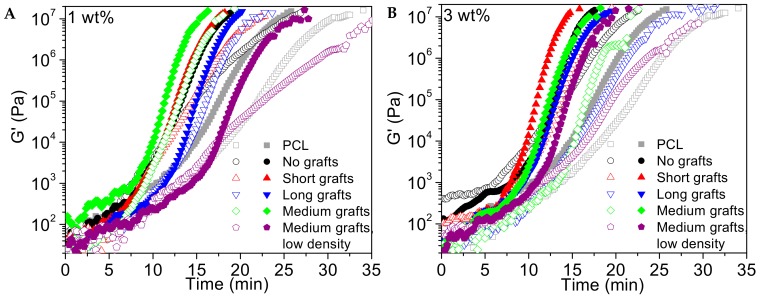
Storage modulus as a function of time for different PCL40/silica composites with 1 wt% filler (**A**) and 3 wt% of filler (**B**). Open symbols corresponds to crystallization experiments without pre-shear and filled symbols to experiments where a pre-shear of 30 s^−1^ was applied for 3 sec. Measurements were performed at a temperature of 47 °C.

Here the development of the storage modulus as a function of time at a certain temperature has been used as a measure for crystallisation and the results are generally in good agreement with other measurement techniques, such as DSC [34]. In Figure 14 the development of the storage modulus as a function of time is presented. The experiments were conducted at 47 °C, which was reached via rapid cooling from the melt, and results of both sheared and non-sheared samples are presented. The filler content was 1 wt%. The nucleation effect of the fillers can clearly be seen in both sets of samples, and the results are consistent with the crystallisation data from the DSC measurements. The non-grafted, short chain length grafted, and medium chain length grafted samples show the fastest modulus build up, while fillers with longer grafts are crystallising slower, though faster than the neat PCL matrix. When comparing the sheared with un-sheared samples it should be noted that the effect of adding non-grafted, short grafted and medium grafted particles is greater than the application of a step shear pulse. If shear is applied to these samples, the crystallisation rate is not largely increased since it is already a relatively fast process. This is consistent with results from other nucleated systems in the literature [35]. For nucleated samples, very high shear rates need to be applied in order to further increase nucleation efficiency, since an important number of nucleation sites are already present. The samples with somewhat longer grafts are crystallising slower than samples with fillers without grafts or with short grafts under quiescent conditions and for these samples the shear impulse is an effective way of enhancing nucleation. As shown in the frequency sweep, the viscosity increase is the largest in samples with the shorter grafts indicating that increased fraction of low molecular weight polymer from the longer grafts is counteracting the effect of the addition of hard silica particles. However, since the shear pulse is strong enough to orient the polymer chains and subsequent relaxation of the formed oriented structures is sufficiently slow, a higher nucleation density and a faster overall crystallisation rate as compared to the quiescent condition is observed. The effect of graft density can also clearly be seen. Particles with the lower graft density are crystalizing very slowly under quiescent conditions and the application of a shear pulse is, therefore, greatly accelerating the crystallization. In samples with a higher graft density the crystallization under quiescent conditions is already quite fast and, therefore, there is less influence of the shear pulse. 

**Figure 15:**
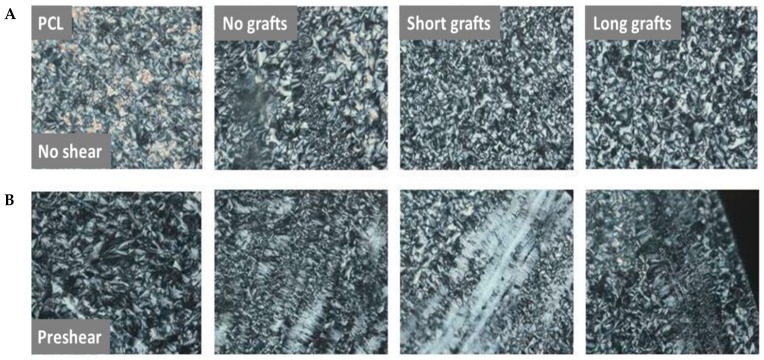
POM images of different samples after crystallisation under different conditions without applied shear (**A**) and with an applied shear of 30 s^−1^ for 3 sec (**B**). Composite samples were based on the PCL40 matrix and contained 1 wt% filler. From left to right: neat PCL, composites with silica particles with no grafts, shorts grafts, long grafts.

Figure 15 shows POM images of PCL40 samples crystallised under different conditions. Clearly, samples subjected to shear show more oriented structures than samples that are crystallised under quiescent conditions. An exception is the neat PCL40 sample, which shows a pure spherulitic morphology also after shearing. The reason for this is that the relaxation time of neat PCL is too short and, therefore, no oriented structures can be formed. The samples with fillers with no or short grafts have longer relaxation times than the neat PCL samples. This allows the samples to retain their orientation after shearing. The samples with the highest viscosity and longest relaxation time during the frequency sweeps were the composites with short grafts, which are also the samples that show the highest orientation after shear.

## 4. Conclusions

Silica nanoparticles with different graft lengths have been prepared via a ring opening polymerisation of ε-caprolactone in the presence of silica. The grafted nanoparticles were mixed into PCL matrices with two different molecular weights (PCL40, 40 kg/mol and PCL80, 80 kg/mol) and their morphology and nanofiller dispersion was investigated. It was shown that the ratio between graft length and the molar mass of the matrix is important for the state of dispersion of the nanocomposites. In the lower molecular weight matrix PCL40, no clear influence of graft length on crystallization temperature was found. Compared to neat PCL40, the rate of crystallisation was improved when short or medium graft lengths were used. An increase in filler content led to higher viscosities and a transition to solid-like rheological behaviour in the low frequency regime. The application of a shear pulse increased the rate of crystallization in samples that showed slow crystallization under quiescent condition but was not effective for samples that already showed enhanced crystallization due to the addition of the nanofiller. However, shear promoted the formation of oriented structures. For the higher molecular weight PCL80 matrix the addition of nanosilica acted as an effective nucleation agent, increasing the crystallisation temperature of the sample already at the very low filler content. Increasing the graft length increased the nucleation efficiency without causing any significant changes in the degree of crystallinity or rheological behaviour. However, for all investigated matrices the non-grafted fillers showed the best nucleation ability, which can be attributed to the already excellent dispersion of non-grafted silica in PCL.

## Figures and Tables

**Table 1:** Description of the different grafting experiments.

Sample Code	Number of NH (groups/nm^2^)	Particles (g)	Catalyst (mg)	Caprolactone (g)	Toluene (g)	Time (h)
Short grafts	0.5–0.7	1.1	100	6.0	80	16
Medium grafts (half density)	0.6–0.8	1.1	67	5.4	90	70
Medium grafts	0.5–0.8	1.1	122	9.9	90	70
Long grafts	0.5–0.8	1.0	118	8.1	95	72

**Table 2:** Weight of the grafted polymers as estimated from TGA data.

Particles	Silanized Particles	Short Grafts	Medium Grafts, Half Density	Medium Grafts	Long Grafts
Est. amount of grafts (% of silica particle weight)	3.8	14.0	15.9	21.5	27.7

**Table 3:** Crystallisation parameters for PCL/silica composites based on PCL40 or PCL80 matrix.

Composite	Silica Content (wt%)	T_m_ (°C)	T_c_ (°C)	Crystallinity (%)	N.E. (%)
PCL40					
PCL40	0	56.3	35.8	47.0	0.0
No grafts	1	56.7	35.8	44.8	−0.3
	3	56.4	36.9	45.4	11.6
	5	56.5	34.4	43.5	−15.4
Short grafts	1	56.6	36.3	47.9	5.5
	3	57.0	36.5	46.2	7.3
	5	56.4	36.6	47.0	8.2
Medium grafts	1	57.2	35.9	43.9	1.3
	3	56.4	35.6	46.7	−2.6
	5	56.4	36.2	46.3	4.1
Medium grafts, half density	1	56.4	34.5	45.8	−14.6
	3	56.7	35.4	45.5	−5.0
	5	56.6	35.1	44.2	−7.7
Long grafts	1	56.3	35.8	47.6	−0.7
	3	56.3	35.4	45.4	−4.2
PCL80					
PCL80	1	56.3	36.4	40.8	0
No grafts	1	56.5	40.9	40.9	32.1
Short grafts	1	56.1	38.4	41.3	13.6
Medium grafts	1	56.8	41.2	39.5	22.5
Medium grafts, Half density	1	56.1	41.4	40.6	27.0
Long grafts	1	56.4	41.0	40.2	25.7
